# Exogenous 24nt siRNAs induce AGO4A-dependent silencing via promoter DNA methylation and H3K9me2 deposition

**DOI:** 10.3389/fpls.2026.1826532

**Published:** 2026-05-26

**Authors:** Arvid Hanke, Laura Schütz, Melanie Walz, Nadja Wunsch, Lenard Kreis, Linus Hohenwarter, Alexandra Baßler, Michele Wassenegger, Gabi Krczal, Aline Koch, Veli Vural Uslu

**Affiliations:** 1 RLP Agroscience GmbH, Neustadt an der Weinstraße, Germany; 2 Center for Organismal Studies, Heidelberg University, Heidelberg, Germany; 3 State Education and Research Center of Viticulture, Horticulture and Rural Development, Institute of Plant Protection, Neustadt/Weinstraße, Germany; 4 Institute for Plant Sciences, Plant RNA Biotechnology, University of Regensburg, Regensburg, Germany

**Keywords:** argonaut, DNA methylation, H3K9me, PTGS (post transcriptional gene silencing), SIGS, siRNA - small interfering RNA, TGS (transcriptional gene silencing)

## Abstract

Transcriptional Gene Silencing (TGS) is an essential process in plants for development, gene regulation, defense against viruses, and genome integrity. TGS is predominantly established by RNA-directed DNA methylation (RdDM), a self-reinforcing mechanism in which sRNAs guide transcriptional suppressors to target genomic loci by sequence complementarity, and methylated DNA in turn facilitates sRNA genesis. Recently, exogenous application of promoter targeting long dsRNAs was associated with promoter methylation without any detectable gene silencing. A plethora of sRNAs of different sizes and types form as cleavage products of precursor dsRNAs such as pre-miRNAs, inverted-repeats, viral replication intermediates, or exogenous dsRNAs. Due to the complexity of sRNA products, the features of the sRNAs, which trigger *de novo* RdDM, remain enigmatic. Here, we demonstrated that *in planta* delivery of chemically synthesized 24-nucleotide(nt) small interfering RNAs (siRNAs), targeting the 35S promoter of GFP-expressing *Nicotiana benthamiana (Nb)* 16c line, was sufficient to induce RdDM, H3K9me2 deposition, and also TGS. Using CRISPR/Cas-mediated gene editing, we showed that exogenous 24nt siRNA-triggered TGS is dependent on ARGONAUTE 4A (NbAGO4A) but not on NbAGO4B. Exogenously administered 24nt siRNAs could provide the means to investigate such initiation events, while allowing functional dissection of siRNA classes and their modifications *in planta*.

## Introduction

RNA interference (RNAi) is a fundamental process in plants, involved in development, defense, and stress response. Small RNAs (sRNA) are pivotal for post-transcriptional gene silencing (PTGS) by targeting mRNA transcript degradation or interfering with their translation (Hamilton et al., 2002; Brodersen et al., 2008). They are also involved in sequence-specific transcriptional gene silencing (TGS) via epigenetic modifications, especially in promoters (Wassenegger, 2005; Matzke et al., 2015). While DICER-LIKE 1 (DCL1) processes pre-miRNAs into mature miRNAs, DCL4, DCL2, and DCL3 cleave dsRNAs, originating from viral replication intermediates, inverted repeats, or RNA-dependent-RNA polymerase (RDR) products, into 21nt, 22nt, and 24nt small interfering RNAs (siRNAs), respectively (Henderson et al., 2006). Predominantly 21-22nt siRNAs are loaded onto ARGONAUTE (AGO) proteins and assemble an RNA-induced silencing complex (RISC), which facilitates PTGS (Baulcombe, 2004; Voinnet, 2009). The RISC executes direct cleavage of target transcripts and/or recruits RDR6, which complements the target strand. This newly produced dsRNA can again be processed into secondary siRNAs of differing sizes, extending beyond the initial sRNA target site in a phenomenon termed transitivity, strengthening the silencing (Vaistij et al., 2002). Thus, a self-amplifying positive feedback loop is formed, leading to efficient PTGS of the target transcript (de Felippes and Waterhouse, 2020). Also, the presence of transitive RNAs indicates an active PTGS mechanism. In plants, PTGS pathways are tightly interconnected with TGS, partially sharing their sRNA machinery (Jones-Rhoades et al., 2006).

Early on, studies on viroids elucidated that RNA-directed *de novo* DNA methylation is based on sequence complementarity (Wassenegger et al., 1994). Later on, it was found that sRNAs of 24-25nt were abundant in heterochromatic regions and were associated with TGS (Hamilton et al., 2002; Zilberman et al., 2003; Cheng and Martinez, 2024). DNA methylation in TGS is often mediated at promoter areas by cytosine-5 methylation (5mC), which exists in three sequence contexts in plants: CG, CHG, and CHH. Once established, the former two can be meiotically and mitotically maintained based on their symmetry, facilitated by the methyltransferases METHYLTRANSFERASE 1 (MET1) for CG and CHROMOMETHYLASE 3 (CMT3) for CHG (Cao et al., 2003; Aufsatz et al., 2004). CHH-methylation, however, cannot be transferred in such a manner, and maintenance requires *de novo* methylation, thus being the hallmark of RdDM (Pélissier et al., 1999). Canonically, RdDM is directed by DCL3-derived 24nt siRNAs, which are AGO4-loaded and guide DOMAINS REARRANGED METHYLTRANSFERASE 2 (DRM2) to its target DNA. Interestingly, most AGO-loaded siRNAs show a strong bias towards one nucleotide in their 5’ position, differing depending on their size, with Adenosine being the most prevalent for 24nt siRNAs (Mi et al., 2008). The AGO-loaded siRNAs typically do not bind complementary DNA directly, but to a scaffold transcript generated by plant-specific RNA Polymerase V (PolV), which is primarily active in heterochromatic regions (Law and Jacobsen, 2010). Likewise, another plant-specific RNA Polymerase IV (PolIV) is primarily active in genomic regions enriched with repressive epigenetic marks, such as transposable elements (TEs), its transcripts are complemented by RDR2, again forming dsRNA substrate for DCL3 to produce further 24nt siRNAs. Once established, the self-reinforcing loop can maintain TGS and is essential to genome stability (Matzke and Mosher, 2014; Zhan and Meyers, 2023). Yet, the initiation of RdDM at a new locus remains puzzling. According to the most recent model, AGO4 clade-loaded 24nt siRNAs are suggested to recruit PolV to its previously unmethylated target locus in a PolII transcript-dependent manner, at least for some TEs (Sigman et al., 2021). Additionally, alternative non-canonical RdDM phenomena have been observed that involve 21- or 22-nt siRNAs, atypical AGO function, and alternative DRM2 recruitment. However, these phenomena remain poorly understood (Wu et al., 2012; Erdmann and Picard, 2020). Besides DNA methylation, histone modifications (and their variants) play a crucial role in epigenetic transcriptional regulation. These two epigenetic processes often reinforce each other, together altering chromatin accessibility. Although many histone modifications with diverging functions exist, here we focus on Histone H3 Lysine 9 di-methylation (H3K9me2), a histone mark commonly associated with DNA methylation, heterochromatin, and transcriptional repression (Ebbs et al., 2005).

High-pressure spraying technique (HPST) is a very powerful tool to modify gene expression *in planta* by initiating PTGS of target transcripts (Dalakouras et al., 2016; Uslu and Wassenegger, 2020; Uslu et al., 2021). Previously, it was also shown that high-pressure spraying of promoter-targeting long dsRNAs led to target site methylation, but not to silencing of the reporter gene (Dalakouras and Ganopoulos, 2021). Because pre-miRNAs and other dsRNAs are processed by DCL into a complex mixture of different sRNA species (Yang et al., 2019; Fard et al., 2020), and because sRNA delivery into intact plants is technically challenging (Bennett et al., 2020), it is nearly impossible to determine the specific contribution of individual sRNAs *in vivo*. HPST enables effective delivery of chemically synthesized sRNA molecules to plant cells in intact plants and allows precise functional dissection of each sRNA in the RNAi pathway *in planta*. Here, we addressed which sRNAs could trigger TGS. We have observed that promoter targeting exogenous 24-nt siRNAs not only led to target-site methylation like sprayed long dsRNAs, but to our surprise, also triggered the silencing of the reporter gene at the transcriptional level, in contrast to long dsRNAs. Having the ability to induce TGS by application of exogenous sRNAs has the potential to pave the road for modern GMO-free epi-breeding strategies (Dalakouras and Vlachostergios, 2021). Furthermore, an on-demand RdDM trigger by a defined siRNA molecule has potential as a research tool, contributing to unraveling and dissecting the complex pathways of RdDM, TGS, and RNAi. In this study, we demonstrated the mode of action of 24nt siRNAs using reporter gene activity, sRNA-sequencing, bisulfite sequencing, chromatin immunoprecipitation, and reverse genetics.

## Materials and methods

### Plant growth and phenotyping

*Nicotiana benthamiana* 16C plants were grown in 25 °C, 16h light - 8h dark cycle. The spraying experiments were performed between 20- and 25-days post germination, corresponding to 4- to 6- true leaf stage. Silencing phenotype was visualized under near UV light (Blak-Ray B-100 AP Lamp, www.uvp.com) and images are obtained by Canon EOS700D (18-55mm) in Aperture Priority Mode (A:10) or Molecular Imager^®^ PharosFX™ Systems (www.bio-rad.com) with GFP and Cy5 fluorescence. The images were overlaid using FIJI (www.imagej.net).

### 22nt- and 24nt siRNA spray

3–4 bar pressure (depending on the leaf size) and 0.5-1bar pressure provided by METABO Elektra Beckum Classic 250 compressor (www.metabo.com) is used for HPST and Low-Pressure (LP) Spray, respectively. Airbrush pistol (CONRAD AFC-250A and CONRAD HP-200) is used to deliver the siRNAs from approximately 1cm distance. 24nt siRNA was provided by Metabion(Germany), 22nt-siRNA is the siRNA164 in Uslu VV et al., 2021 (Uslu et al., 2021). The RNA was dissolved in water for naked RNA spraying at high pressure and Silwett L77 + CD Spray buffer (10mM MES, 20mM glycerol, pH:5.7) is used for CD spray.

### RNA extraction and small RNA sequencing

At 6dps, silenced areas were excised from 2 leaves, showing silencing and were pooled per biological replicate. Three biological replicates of each treatment (approximately 50mg pooled leaf material) was crushed using metal beads and small RNAs were extracted using mirVana miRNA extraction kit according to manufacturer’s instructions (www.thermofisher.com). sRNA-seq, quality control and adapter trimming were performed by GenXPro, Frankfurt (http://genexpro.net) as indicated in the literature (Uslu et al., 2021).

### Bisulfite sequencing

Genomic DNA is extracted with the MACHEREY-NAGEL Plant II Kit (www.mn-net.com) using buffer system PL1, and bisulfite conversion of DNA is performed using Zymo EZ DNA Methylation-Lightning™ Kit (www.zymoresearch.de), both according to manufacturer’s instructions.

35S promoter PCR template is converted by bisulfite kit to evaluate conversion efficiency (>99%). DreamTaq DNA polymerase (www.thermofisher.com) is used to amplify the bottom strand with the following primer pair:

1995_24nt_35Sp_oppStr-Fwd: 5’-TCCCAAARATRRACCCCCACCCAC-3’,

1996_24nt_35Sp_oppStr-Rev: 5’-TGAAAAGATGAGAAAGAGAAAAAGATTAG-3’.

The PCR products after 40 cycles are cloned into the sequencing vector of the CloneJET PCR Cloning Kit (www.thermofisher.com). T7 primer is used for sequencing the insert. Initial analysis of methylation has been performed by CyMATE (www.cymate.org) and the data is further curated to exclude sequencing errors manually.

### Fluorescence quantification

Fluorescence values determined in Molecular Imager^®^ PharosFX™ generated images were measured in Fiji (Schindelin et al., 2012), then normalized each value to the mean of non-sprayed area fluorescence, as fluorescence varies between leaves and between the vasculature and the rest of the leaf.

### Chromatin immunoprecipitation

500 mg leaf material without main vasculature per sample was homogenized to a fine powder in liquid nitrogen. The homogenate was directly cross-linked in formaldehyde and nuclei extracted according to Ye et al., halving the volumes to accommodate for the smaller amount of input material (Ye et al., 2022). The nuclei pellet was suspended in 300 µl Nuclei lysis buffer supplemented with fresh Halt™ Protease-Inhibitor-Cocktail (www.thermofisher.com) and sonicated with a MISONIX sonicator^®^ XL2020 (www.bioventus.com/misonix/) to a size of 150-400bp. According to Ranawaka et al. (2020) (Ranawaka et al., 2020), taking 1% input samples, ChIP using 2 µg H3K9me2 antibody Diagenode C15410060 per sample, reverse crosslinking, and DNA purification was performed. Relative enrichment was determined using ChIP-qPCR and the percent input method, using *Ef-1a* (active locus) and Ty1-copia (repressed locus) primers (Ranawaka et al., 2020) to assess ChIP success, eliminating samples Input% Ty1-copia/Input% *Ef-1a* with a ratio smaller than three. Target region CaMV35S promoter-enrichment (primers 35sP_2538_fw: 5’-GGATTGATGTGATATCTCCACTGACG-3’ and 35Sp_2539_rv: 5’-CCCCGTGTTCTCTCCAAATG-3’) was normalized to the active genes (Haring et al., 2010) (Input% CaMV35SP/Input% *Ef-1a*).

### qPCR and cDNA synthesis

All qPCRs were performed using Luna^®^ Universal qPCR Master Mix (www.neb.com) using 1µl template in technical duplicates according to the manufacturer’s instructions. RNA for cDNA was extracted using TRIzol™ Reagent (www.thermofisher.com) together with phenol-chloroform extraction and ethanol precipitation. After DNAseI digest and re-purification by Monarch^®^ Spin RNA Cleanup Kit (50 μg) half-volume cDNA synthesis was performed with a ProtoScript^®^ First Strand cDNA Synthesis Kit (www.neb.com). GFP transcript was amplified using primers 5’-AAGACCCGCCACAACATCGAAG-3’ and 5’-TCGAAAGGGCAGATTGTGTGGAC-3’ normalized to UKN1 transcript (Hendrix et al., 2021).

### Statistical and sequencing analysis

sRNA-sequencing reads were mapped to the 16c T-DNA sequence (Philips et al., 2017) using the standard settings of BBMap v.39.06 (Bushnell, 2014). A region 460bp bases upstream 1060bp downstream of the GFP start-codon was further analysed, eliminating siRNA sequences sprayed in this and concurrent experiments to exclude exoRNAs stuck to leaf surfaces as previously reported (Uslu et al., 2021). Mapped reads after filtering were normalized to total reads (reads per million reads, RPM) to facilitate library comparison. Statistical analysis and graphing were performed in custom R v. 4.5.1 scripts (R Core Team, 2025).

## Results

### HPST of 24nt but not 22nt siRNAs induced transcriptional gene silencing *in planta*

**Figure 1:**
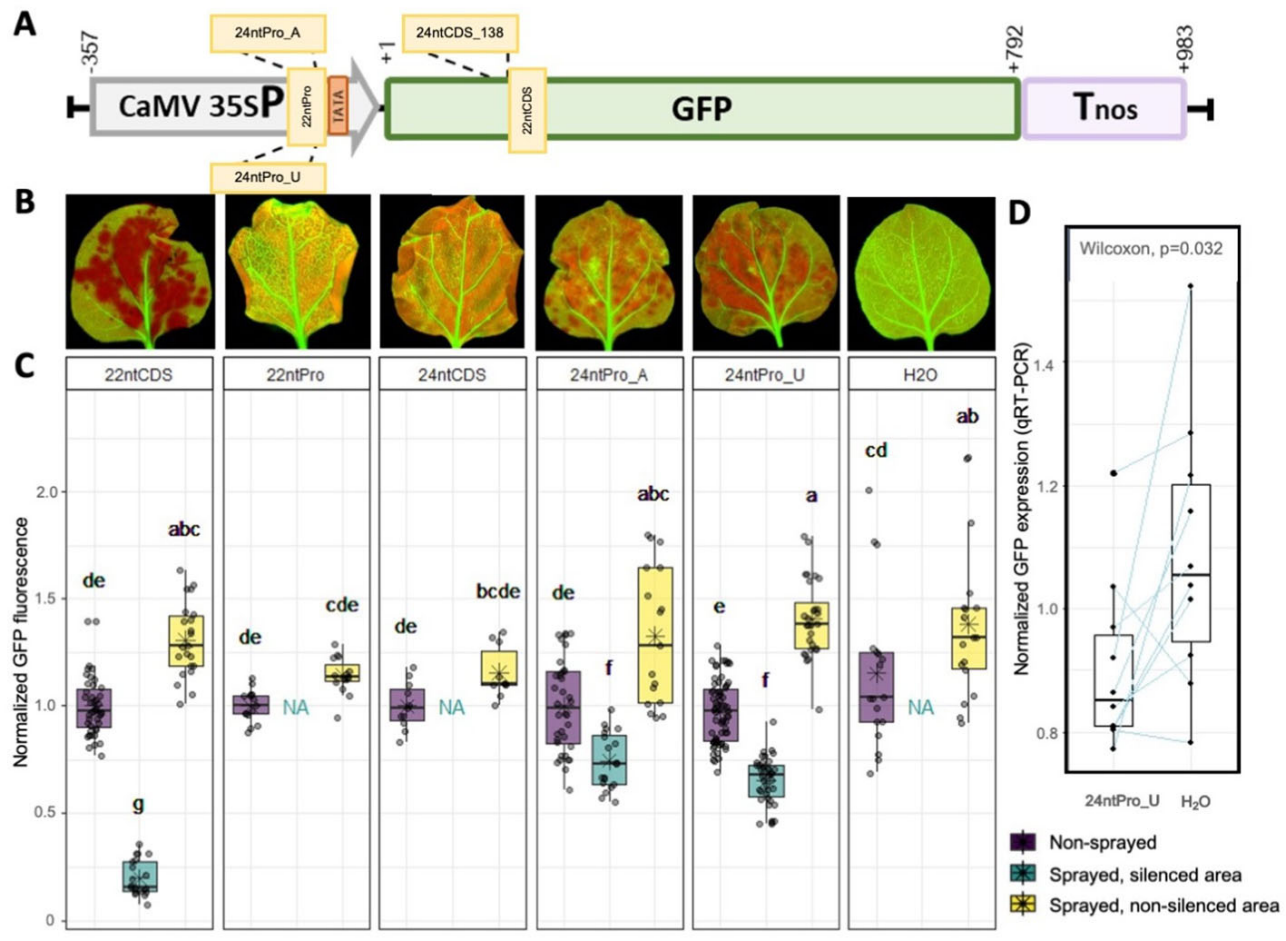
Local *Nb* 16c GFP silencing phenotype of sprayed siRNAs. **(A)** 16c GFP locus, promoter TATA box (red) and siRNA target sites (in orange) are indicated with siRNA length and target (CDS=GFP coding sequence, Pro= CaMV 35S promoter). **(B)** Representative *Nb* 16c leaves 10 days after siRNA (or H2O) high-pressure spray application. False colors; green=GFP channel, red=far red for chlorophyll auto-fluorescence. **(C)** Boxplot of GFP intensity of minimum of 6 measurements from two leaves and three repetitions, each for leaf sectors that were not sprayed (purple), sprayed and show silencing (petrol), and sprayed (and partly wounded) but show no silencing (yellow) *= mean, Letters: ANOVA p<0.05. **(D)** Expression reduction of GFP by qRT-PCR of 10 leaf halves sprayed with siRNA, or H_2_O, paired Wilcoxon-test pairing respective halves.

In order to investigate 24nt siRNAs’ potential in triggering RdDM and inducing TGS, we designed synthetic HPLC purified siRNAs of different lengths, types, and complementarity (**Supplementary Table 1**). They were designed to target the *Nb* 16c reporter lines CaMV35sP::GFP:tNOS cassette (**Figure 1A**), and were applied via HPST (Dalakouras et al., 2016). We designed a 24nt siRNA, targeting the most CG and CHH dense sequence in the 35S promoter, slightly upstream of TATA-box with a 5’A (24ntPro_A) to test the role of canonical 24nt siRNAs in TGS pathway. Besides, we also designed another 24nt siRNA with a 5’U (24ntPro_U), with a large overlapping sequence with 24ntPro_A, to investigate the importance of the 5’ nucleotide. We also designed a 22nt siRNA, targeting the 35S promoter (22ntPro), which was implicated in non-canonical transcriptional gene silencing. As a negative control, we designed a CDS-targeting 24nt siRNA (24ntCDS_138), together with water spraying. The GFP CDS was also targeted by HPST of 22nt siRNA (22ntCDS), which yielded strong silencing based on the previous results and served as a positive control in this study (**Figure 1A**) (Uslu et al., 2021).

Silencing in the *Nb* 16c reporter system is visualized as red chlorophyll auto-fluorescence in the absence of green GFP fluorescence under near-UV excitation. Red silencing spots emerged in *Nb* 16c plants, the positive control, where 22ntCDS was sprayed, as early as 4 days post-spraying (dps) (**Figure 1B**). On the other hand, 24ntCDS_138 was negative for the silencing phenotype, similar to water control, as expected (**Figure 1B**) (Dalakouras et al., 2016). 22ntPro also failed to induce any detectable TGS (**Figure 1B**). Yet, 24ntPro_A and 24ntPro_U treatments led to detectable GFP silencing starting from 5dps. Compared to the strong PTGS previously reported for 22ntCDS HPST (Uslu et al., 2021), the phenotype in 24ntPro_A/U-treated plants was milder, showing an intermediate orange fluorescence (**Figure 1B**). Additionally, while silencing in 22ntCDS samples spread over time and became systemic, silencing in 24ntPro-treated *Nb* 16c plants peaked around 10dps, gradually dissipated as the leaves grew, and never spread systemically (**Supplementary Figure 1**).

Quantification of GFP fluorescence by image analysis (**Figure 1C**) confirmed our initial visual observations. Notably, GFP signal increased slightly after spraying regardless of treatment, even in H_2_O-sprayed and non-silenced areas presumed to originate from auto-fluorescent wounded tissues, as previously suggested (Haseloff and Siemering, 2006). Despite this background effect, strong GFP reduction was observed following 22ntCDS HPST, while 24ntPro_A and 24ntPro_U HPST caused intermediate reduction (**Figures 1B, C**). As no significant differences were detected between the two 24ntPro variants, subsequent experiments were only performed using 24ntPro_U. qRT-PCR analysis of GFP transcript levels (**Figure 1D**) aligns with the fluorescence data, showing a significant decrease in GFP mRNA in 24ntPro_U-treated leaf halves compared to the corresponding H_2_O-sprayed halves. Previous studies indicated that carbon dots (CD) could act as efficient carriers for 21nt and 22nt siRNAs, facilitating efficient PTGS even with gentle, low-pressure spraying (Schwartz et al., 2020). We thus implemented CD-based formulation of 24ntPro_U to improve the efficacy of silencing. However, we observed no silencing in three independent experiments, whereas 22nt siRNA controls (HPST/low-pressure CD) induced efficient silencing as previously reported (**Supplementary Figure 2**).

### Promoter targeting 24nt siRNAs yield longer RNAs around the target site in the promoter, but not secondary RNAs in the coding area

**Figure 2:**
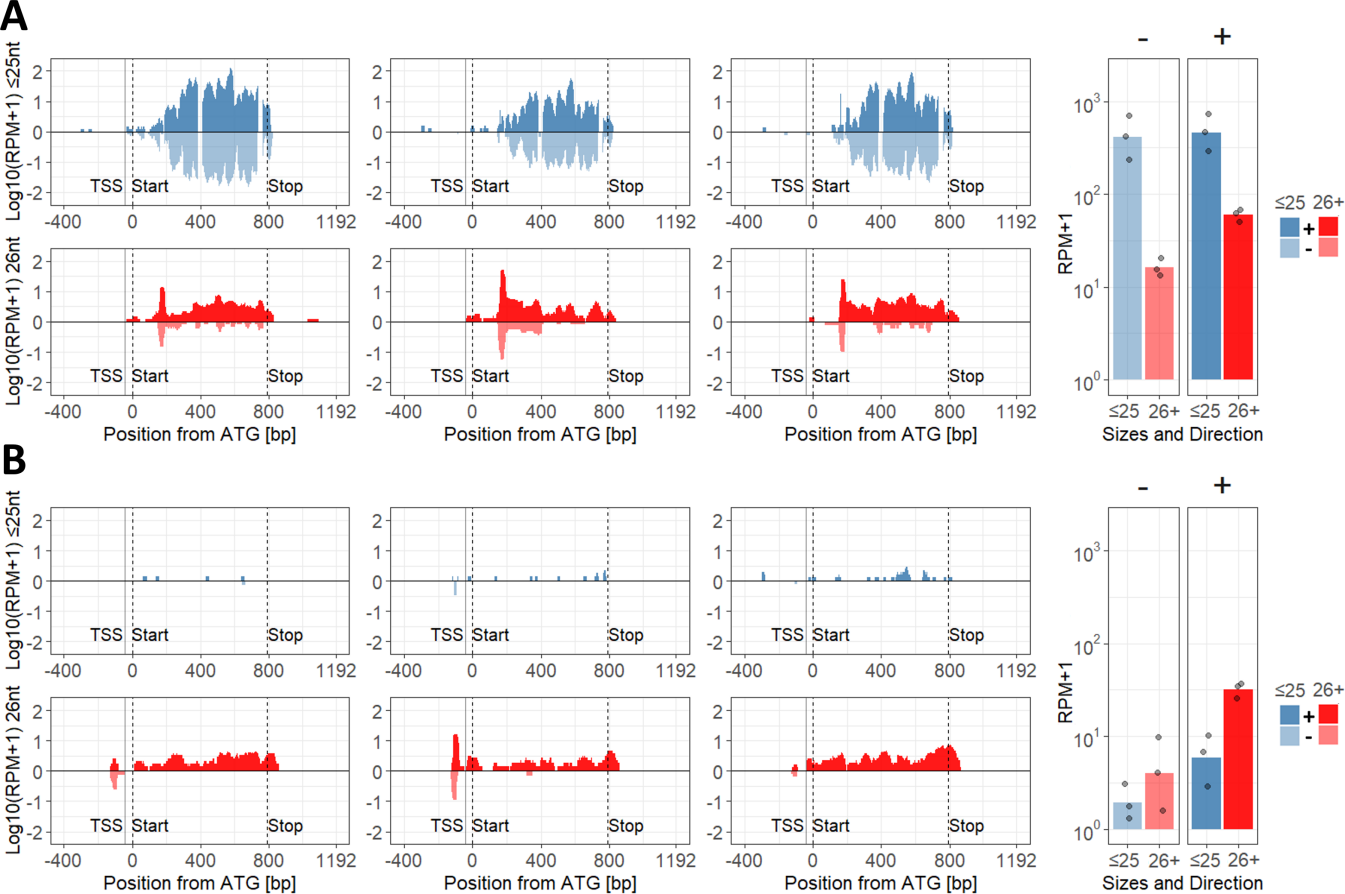
Small RNA profile after 24ntHPST diverges from 22nt HPST induced PTGS profile. Reads per million (RPM) mapping to the *Nb* 16c GFP target transgene 460bp upstream and 1040bp downstream of start codon in sense (+) and antisense (-) direction, 6 days after HPST. CDS Start & Stop and transcription start site (TSS) indicated. **(A)** 22ntCDS and **(B)** 24ntPro_U. 3 biological replicates each, subdividing reads by their length and summarizing replicates in the rightmost panel. Applied siRNA sequences of this and concurrent experiments were filtered to avoid leaf surface contaminations. In each panel, red and blue signals above the X-axis indicate sense strand reads, whereas lighter-colored reads below the X-axis indicate anti-sense reads.

Previously, 24nt siRNAs were associated with RdDM and TGS, based on the absence of RdDM phenotype in the absence of 24nt siRNAs in *dcl3* mutants, or the presence miRNAs, which are predominantly but not exclusively cleaved into 24nt sRNAs (Xie et al., 2004; Henderson et al., 2006; Daxinger et al., 2009; Wu et al., 2010; Melnyk et al., 2011; Sablok et al., 2015; Fard et al., 2020). Such data was not enough to exclude a potential role of 24nt siRNAs as a PTGS-trigger, along with their role in TGS. To further elucidate the mode of action underlying the observed silencing, sRNA sequencing (sRNA-seq) was performed on 24ntPro_U HPST-treated leaves 6dps, and compared to previously reported analogous 22ntCDS sRNA-seq data (**Figure 2**) (Uslu et al., 2021). Previously published libraries reflected typical 22ntCDS HPST-induced PTGS, showing pronounced transitivity and secondary siRNA formation in both sense and antisense direction along the target transcript, particularly siRNAs of 20-25nt length (Uslu et al., 2021). Transitive siRNAs also don’t extend beyond the transcription start site (TSS), determined by 5’ RACE to lie between -42 and -38 from translation start. In contrast, libraries obtained from leaves after 24ntPro_U HPST treatment (**Figure 2B**) differ strongly from these PTGS-associated signatures. Only very few siRNAs map to the target transcript, and these map almost exclusively to the sense strand, similar to the previously published sRNA-sequencing of the water-sprayed samples (**Supplementary Figure 3**) (Uslu et al., 2020). Likewise, longer RNAs are found predominantly in the sense direction, except around the 24ntPro_U target region itself, where promoter-mapping reads longer than 25nt accumulate in both sense and antisense direction. These promoter-derived longer transcripts are absent in the 22ntCDS-treated samples; their siRNA target locus, however, also shows an accumulation of 26nt+ RNAs (**Figure 2**, **Supplementary Figure 4**). These features, the lack of antisense transitivity, the minimal production of typical PTGS-associated 21–22nt siRNAs, and the accumulation of longer promoter-mapping RNAs are inconsistent with canonical PTGS. Instead, they suggest that 24ntPro_U triggers a distinct, potentially TGS-associated silencing mechanism as proposed in previous studies (Henderson et al., 2006; Daxinger et al., 2009; Wu et al., 2010; Melnyk et al., 2011).

### 24nt siRNAs induce RdDM and deposition of repressive histone methylation at and around the target site

**Figure 3:**
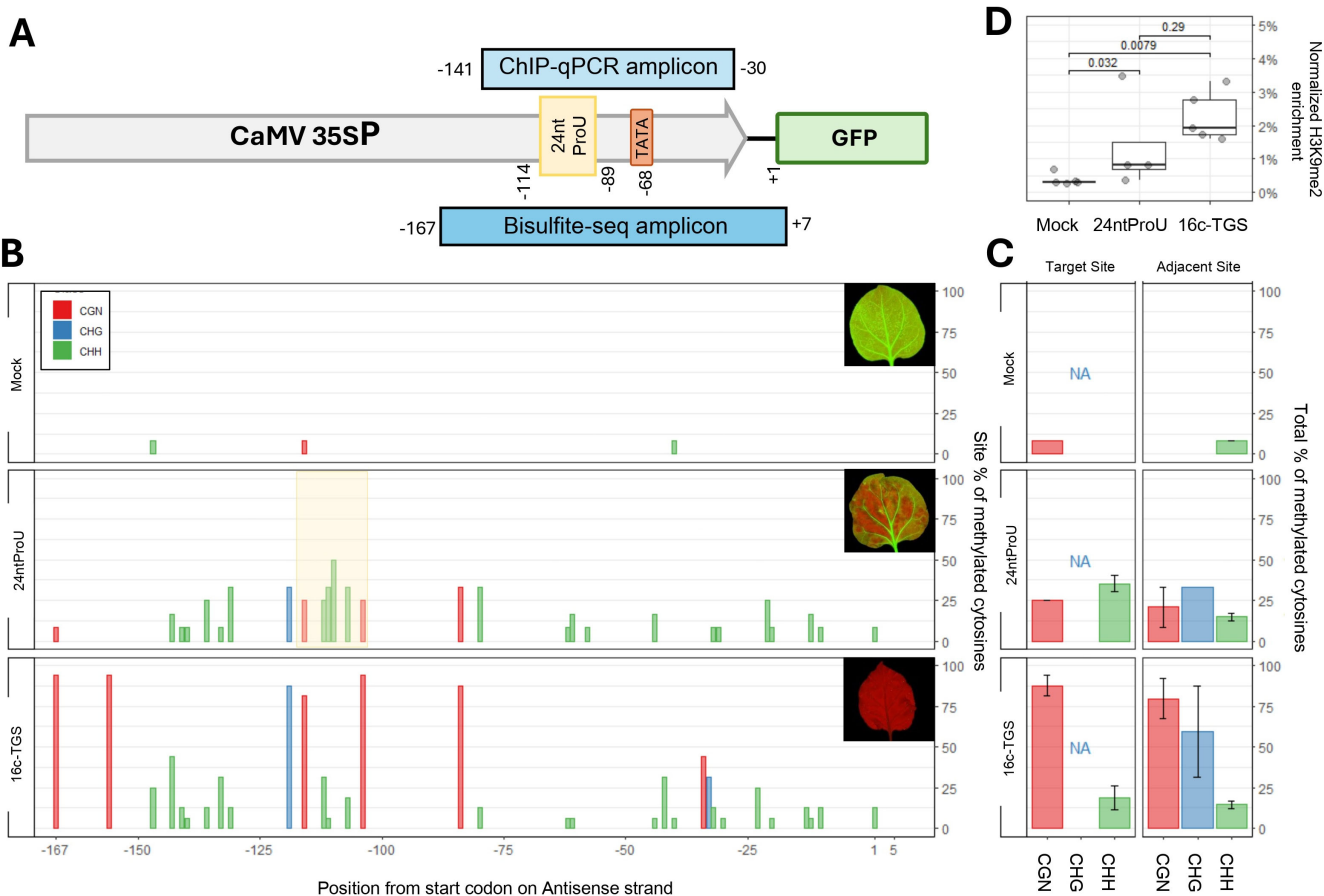
CaMV35Spro complementary 24nts HPST induces epigenetic changes around target site **(A)** Schematic of the 16c reporter transgenes CaMV 35S promoter with 24nt siRNA target site, TATA-box, ChIP-amplicon site and area analyzed for 5-Methylcytosine DNA methylation (5mC) in **(B, C)**: Bisulfite-seq demonstrates low basal 5mC level in mock sprayed samples, and a strong increase in 5mC upon 24nt siRNA application, especially at and adjacent of the highlighted target site. Stably silenced 16c-TGS line exhibits high 5mC, especially in symmetrical CGN and CHG contexts. Mean 5mC % depicted represents N = 12–16 unique Sanger-reads per condition from three biological replicates, color-coded by cytosine context. Whiskers depict standard error. **(D)** H3K9me2 ChIP-qPCR of the promoter shows stat. significant signal increases over untreated 16c control in both 24nt siRNA-treated and stably silenced 16c-TGS samples after immunoprecipitation, p-values for Wilcoxon rank-sum test, N = 4-6.

After excluding any PTGS-associated signatures, we next examined whether GFP downregulation correlated with the induction of TGS-associated epigenetic marks upon 24nt-siRNA spraying. For this, we performed bisulfite-sequencing and chromatin immunoprecipitation (ChIP) analyses at and around the siRNA target site (**Figure 3**). Cytosine methylation in the analyzed *Nb* 16c GFP promoter region is absent in H2O-sprayed controls, consistent with a previous report (Dalakouras and Ganopoulos, 2021). As a positive control for DNA methylation, we used a *Nb* 16c-derived line known to exhibit stable TGS, here referred to as 16c-TGS. In this *Nb* line, GFP silencing was originally induced by a virus-induced gene silencing (VIGS) approach, and behaves as a stable epi-allele even in the absence of the inducing virus (**Supplementary Table 3**) (Jones et al., 2001). As expected, 16c-TGS samples displayed exceptionally high 5mC in the symmetrical CG and CHG contexts, exceeding 80% upstream of the TATA-box and decreasing towards the start-codon. Because the 16c-TGS line is not targeted by the 24ntPro_U siRNA, the DNA methylation patterns were the same between the siRNA mapping region and the adjacent region. Ten days after 24nt_U application, 5mC strongly increased in the examined promoter region, especially at the siRNA target site, where levels reached up to 50% 5mC (**Figures 3B, C**). Adjacent sites also showed detectable, but lower, methylation levels. Total methylation was similarly distributed across symmetrical and asymmetrical contexts. Notably, a small number of sequence reads accounted for the majority of detected 5mC, whereas most reads remained largely unmethylated (**Supplementary Figure 5**). It is a pattern, which is also observed in promoter-targeting long dsRNA spraying (Dalakouras and Ganopoulos, 2021). The lack of clear evidence for *de novo* synthesized dsRNAs in the promoter in sRNA-seq (**Figure 2**), rules out substantial amplification of sRNA by RDR2 (Llave, 2010). Therefore, the silencing phenotype can only be observed in those cells, to which 24nt siRNA is successfully delivered and transported to the nucleus. Also considering that different AGO proteins are differentially active in different cell types in the shoot apical meristem in *A. thaliana* (Nguyen and Gutzat, 2022), we cannot rule out that some cell types in the leaf process the delivered RNAs differently. It is noteworthy that the 22ntCDS-mediated silencing is observed in local vascular tissue, but we have never seen vascular silencing with 24ntPro_A or _U (**Figure 1B**). Therefore, it is plausible to hypothesize that the mosaicism in the methylation marks may both reflect the cells that were successfully targeted by spraying and the cells with distinct AGO repertoires. Investigating another repressive epigenetic mark, H3K9me2, by ChIP-qPCR 10dps revealed increased enrichment in both 24ntPro_U-treated and the 16c-TGS positive controls, compared to untreated *Nb* 16c leaves (**Figure 3D**). These results show that HPST-delivered, promoter-complementary 24nt siRNAs are sufficient to induce two hallmark repressive epigenetic modifications: cytosine methylation and H3K9me2 deposition.

### 24nt siRNA-mediated transcriptional gene silencing is controlled by AGO4A but not by AGO4B

**Figure 4:**
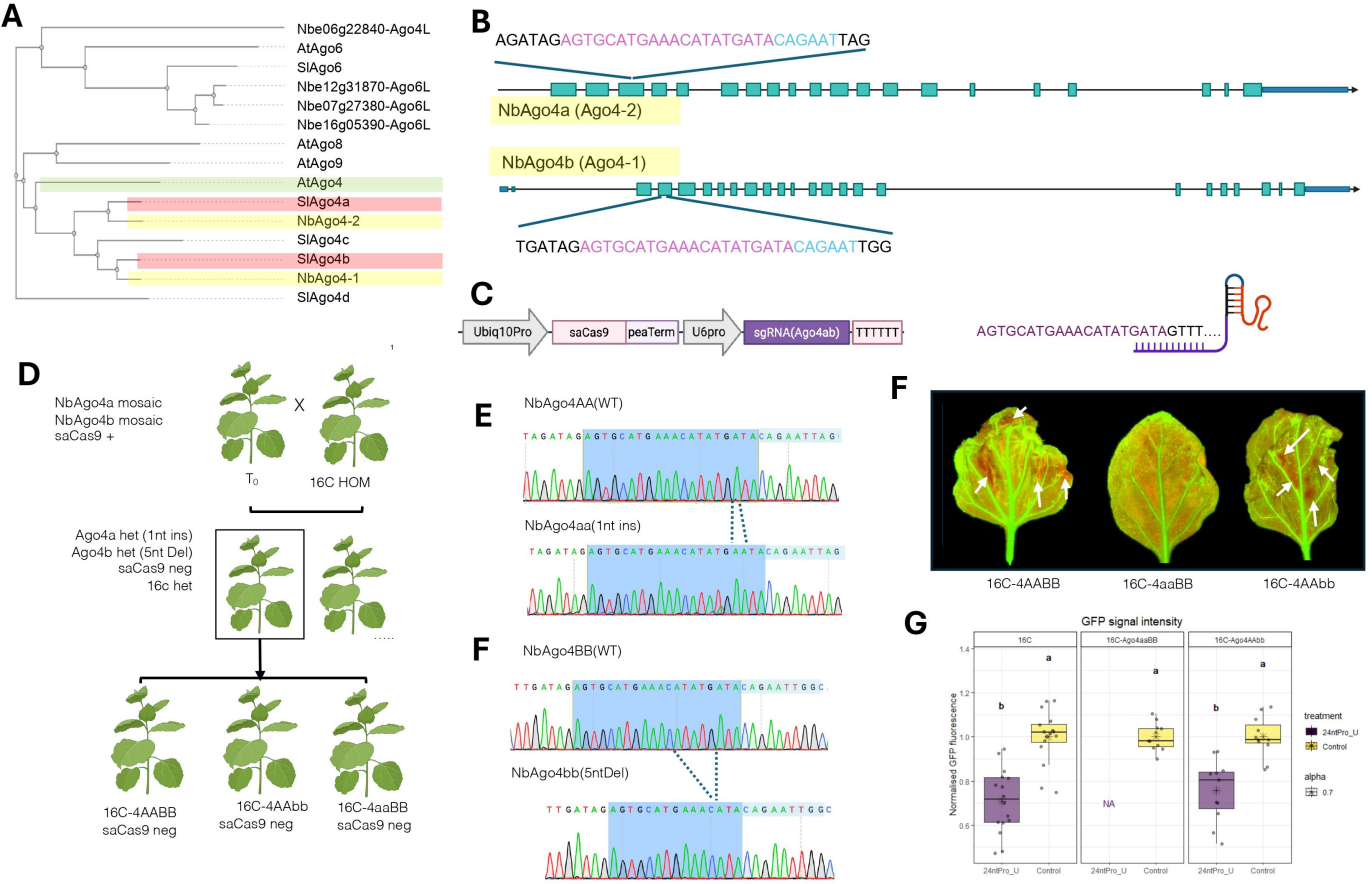
CRISPR-Cas mediated knockout of AGO4 homologs in *Nicotiana benthamiana*. **(A)** The ClustalW treeview of TGS-associated AGO proteins in At, Nb, and tomato shows that NbAGO4A and NbAGO4B are ancestrally related to AtAGO4. NbAGO4A was previously labeled as NbAGO4-2, and NbAGO4B was previously labeled as NbAGO4-1. **(B)** The schematic representation of the *NbAgo4a* and *NbAgo4b* genes and the common 20nt sequence sgRNA target site is shown in lila, and the PAM sites are shown in blue font in exon 3 and exon 4, respectively **(C)** The schematic representation of the Cas9-sgRNA expression vector is given. saCas9 expression is controlled by *Arabidopsis thaliana* (*At*) Ubiquitin10 promoter, and the sgRNA expression is under the control of *At*U6 promoter **(D)** GFP-expressing *NbAgo4a* and *NbAgo4b* mutant lines were obtained after crossing with *Nb* 16c plants and segregating out the saCas9. T3 generation was screened for *Ago4a* and *Ago4b* mutations by sequencing and 16c-reporter homozygosity by evaluating the distribution of GFP-expressing plants. **(E)** *Ago4a* mutation (NbAgo4aa) was a homozygous single-nucleotide insertion, and **(F)** *Ago4b* mutation (NbAgo4bb) was a homozygous five nucleotide deletion. We obtained GFP homozygous-16C-NbA4AABB non-mutated Nb plants, 16C-Nb4AAbb mutant, which lacks *NbAgo4b* and has a fully functional *NbAgo4a*, and 16C-Nb4aaBB, which lacks *NbAgo4a* and has a fully functional *NbAgo4b*. **(G)** 24ntPro_U spraying on 16C-4AABB (WT) and 16C-4AAbb led to silencing (white arrows), whereas 16C-4aaBB did not show any silencing (N≥3 for each genetic background).

It was previously shown that 24nt siRNAs, which were processed by DCL3, are loaded mostly onto AGO4, but in specific cases, also onto AGO6 or AGO9 in Arabidopsis (Duan et al., 2015). Therefore, we hypothesized that 24ntPro_U was loaded onto AGO4 homologs in *Nb* and silenced the 35S promoter in the 16c reporter line. Based on previous literature and ClustalW amino acid sequence comparison of AGO homologs in Nb, we found that NbAGO4–2 and NbAGO4–1 shared the highest homology to AtAGO4, and they were named as NbAGO4A and NbAGO4B, respectively, based on their similarity to tomato SlAGO homologs (**Figure 4A**). We aligned *NbAgo4a* and *NbAgo4b* coding sequences and found an identical 20nt target site in the 3^rd^ and 4^th^ exon, respectively, with an adjacent CAGAAT PAM site, suitable for *Staphylococcus aureus* (*Sa*) CAS9 (**Figure 4B**). To test the function of NbAGO4 on 24nt exoRNA-mediated gene silencing, we designed a *SaCas9* expression construct under the control of *A*rabidopsis ubiquitin-10 (AtUbiq10) promoter, targeting the shared sequence (**Figures 4B, C**). The single guide RNA (sgRNA) was expressed using an AtU6 promoter, and the selection marker was hygromycin-resistance (HygroR+) (**Figure 4C**). After leaf disk transformation, HygroR+ shoots were selected and genotyped for *SaCas9*, *NbAgo4a*, and *NbAgo4b* (**Figure 4D**). Approximately twenty shoots positive for *SaCas9* and mosaic for *NbAgo4a* or *NbAgo4b* sequences were transferred to soil, and their flowers were used in reciprocal crossing with 16c GFP-homozygous plants. The next generation (T1) was heterozygous for GFP, and we screened for Cas9-negative plants with mutations in *NbAgo4a* and *NbAgo4b*. We detected heterozygous *NbAgo4a* mutations with 1nt insertion and heterozygous *NbAgo4b* mutations with 5nt deletion, which led to frameshift in both cases. Seeds of these plants were collected to establish stable homozygous mutants. First, we selected GFP-positive 16c (heterozygous or homozygous), and we further genotyped the plants for homozygous *NbAgo4a* mutants with wild-type *NbAgo4b* (Nb4aaBB) and homozygous *NbAgo4b* mutants with wild-type *NbAgo4a* (Nb4AAbb). The saCas9 genotyping PCR was performed on the T2 generation to avoid any possible mistakes, and the plants were confirmed for the absence of saCas9. In T3 generation, the GFP homozygosity of 16c was verified by the distribution of GFP-expressing plants, and the 1nt homozygous insertion in *NbAgo4a* (**Figure 4D**) *NbAgo4a* deletion, and 5nt homozygous deletion in *NbAgo4b* were verified by sequencing (**Figure 4E**). Nb4aaBB and Nb4AAbb plants did not show a visible growth or developmental phenotype, similar to what was observed in *NbAgo4* knockdown (Jones et al., 2006). 24ntPro_U was sprayed by HPST onto the Nb4AAbb, Nb4aaBB mutants and Nb4AABB plants. The silencing phenotype was detected in Nb4AAbb, and Nb4AABB, but it was completely abolished in Nb4aaBB (**Figure 4F**). Quantification of the GFP signal demonstrated that the level of silencing in Nb4AAbb, and Nb4AABB was not statistically different (**Figure 4G**). These results suggested that NbAgo4a is essential for exoRNA-mediated TGS but not NbAgo4b.

## Discussion

### The dissipation of 24nt siRNA-mediated silencing over time

We demonstrated that high-pressure spray application of a 24nt siRNA targeting the CaMV35S promoter of the *Nb* 16c GFP reporter line induces local TGS, which does not spread systemically and is notably weaker than the PTGS previously reported when the same reporter line was targeted at the GFP-CDS with 22nt siRNAs (Dalakouras et al., 2016). Rather, the intermediate orange fluorescence and weak but statistically significant reduction in GFP transcript abundance indicate only partial silencing, even within directly affected leaf areas (**Figure 1**). This TGS phenotype peaks around 10dps and subsequently declines within weeks (**Supplementary Figure 1**). Two mechanisms could explain this reversibility. First, expanding leaf tissue may undergo mitotic divisions that dilute DNA methylation marks in the absence of a continuous siRNA trigger. Second, the CaMV35S is a strong, actively marked promoter enriched in H3K4me3, an active histone mark, recently shown to recruit DNA demethylases for active removal of the cytosine methylation (Wang et al., 2025). Such promoter-intrinsic chromatin features may therefore counteract and limit the stability of the 24ntPro_U-induced TGS.

### Potential reasons for the different silencing phenotypes of dsRNA spraying and sRNA spraying

Recently, the first example of spray-induced epigenetic modifications (SPIEM) has been reported, where HPST of a 333bp dsRNA induced CaMV35S promoter methylation 10dps in *Nb* 16c, although without detectable target gene downregulation (Dalakouras and Ganopoulos, 2021). We previously demonstrated that HPST-delivered siRNAs trigger PTGS substantially more effectively than dsRNA (Uslu et al., 2020). Several factors may explain the differing outcomes between dsRNA- and 24 nt siRNA-based SPIEM: First, although differences in uptake efficiency between a 333-bp dsRNA and 24nt siRNA should be considered, this explanation seems unlikely, as both treatments resulted in comparable 5mC levels. Second, long dsRNAs may enter RdDM pathway differently than chemically synthesized 24nt siRNAs, leading to heterogeneous siRNA populations (Dalakouras and Wassenegger, 2013; Dalakouras and Papadopoulou, 2020). Third, and most plausible, the target position differs. The 24nt siRNA targets a region selected because of high GC content, and its proximity to the transcription start site, whereas the 333-bp dsRNA targeted an upstream promoter region. This positional difference may explain why strong promoter methylation in the dsRNA study did not result in transcriptional repression. However, these findings underline the importance of precise RdDM target site selection and future potential for improvement. Notably, 5mC deposition after 24ntPro_U application was enriched at the siRNA target site but not sharply restricted to it; 5mC also spread beyond the complementary region, indicating that even low CG content (such as the TATA-box) could be considered as potential targets in further development of the technique. Average GFP signal reduction after HPST of 24ntPro was weaker than in 22nt HPST-induced PTGS, with silencing emerging in a spotty pattern and resulting in intermediate orange fluorescence under UV excitation (**Figure 1**). While the current SPIEM protocol is sufficient to investigate the onset of RdDM and TGS in general, proved by detectable *de novo* DNA methylation and H3K9me2 deposition, its efficiency remains poor for potential application as an epigenetic breeding tool. However, improvements to HPST parameters, including handling, are necessary to enhance both coverage and stability of 24nt-induced TGS.

### The role of 5’ nucleotide identity in TGS

Initially, we performed experiments including two 24nt siRNAs that targeted the same locus of the CaMV35S promoter, with either an A or U residue at the 5′ end (24ntPro_A and 24ntPro_U). However, as HPST of both variants resulted in comparable silencing, we pursued subsequent experiments with only one variant, 24ntPro_U, which was selected randomly. AGO4-bound 24nt siRNAs in plants disproportionately contain a 5′ A compared to other nucleotides, which we also address here. Although AGO4 reportedly has a slight preference for a 5′ A (Liu et al., 2022), our results demonstrate comparable silencing regardless of 5’ nucleotide, which is consistent with the current understanding that the abundance of 5’ A 24nt siRNAs also comes from a preference of DCL cut sites and substrate genesis (Vaucheret and Voinnet, 2024) and AGO4 does not dramatically distinguish 5’A or 5’U sequence.

### sRNA-sequencing profile differences between 22nt- and 24nt siRNAs spraying

The 24nt siRNA-induced silencing phenotype differed from previously reported PTGS, triggered by 22nt siRNAs. The canonical roles of siRNA sizes detailed above, where 22nt siRNAs typically induce PTGS and 24nt siRNAs are predominantly associated with TGS, encouraged us to investigate the underlying mode of action. Comparing sRNA-seq profiles of 24ntPro_U treated areas to our previously published 22ntCDS data (Uslu et al., 2021) displayed a striking difference in siRNA signatures (**Figure 2**, **Supplementary Figure 4**). The characteristic PTGS hallmarks present in the 22ntCDS data of transient siRNAs, mostly within the coding region in both sense and antisense direction, along with RDR6-dependent longer antisense transcripts (Hamilton and Baulcombe, 1999; Hendrix et al., 2021; Brosnan et al., 2026) were absent in 24ntPro_U samples. Instead, we observed an accumulation of longer 26-40nt reads around the promoter target site after 24ntPro_U-treatment. These longer RNAs are consistent with typical PolV-derived transcript lengths (Blevins et al., 2015), and further support that the observed silencing is most likely mediated by a TGS/RdDM-type mechanism rather than PTGS.

### The range of DNA methylation spread by 24nt siRNAs

Previous *in vivo* generation of RdDM triggering siRNA from longer dsRNAs, whether selectively expressed within the organism (Přibylová et al., 2019) or exogenously delivered (Dalakouras and Ganopoulos, 2021), results in a heterogeneous population of DCL-processed siRNAs. In contrast, delivery of synthetic siRNAs of a defined structure (and potentially defined modification) allows us to dissect their functions separately. Interestingly, both studies report a sharp methylation cutoff with 5mC almost exclusively established within the target region itself, while in our 24nt siRNA HPST approach, DNA methylation spreads beyond the siRNA target sequence (**Figure 3**, **Supplementary Figure 5**). Přibylová et al. report the absence of transitive siRNAs outside the target, while secondary siRNAs inside the target would be indistinguishable from primary ones. Since we applied only a single siRNA of known sequence, it can be computationally filtered out, leaving remaining reads that map to the target region and adjacent sequences that are likely to originate from transcription of that very locus. The 26nt+ RNAs emerging around the target locus upon 24ntPro_U-treatment (**Figure 2**, **Supplementary Figure 4**) also correlate with the observed spreading DNA methylation. This may suggest mechanistic differences compared to the earlier dsRNA-based approaches. However, the nature of the 26nt+ RNAs requires further investigation, especially to determine whether they represent functional PolV products, required for RdDM initiation.

In addition, the siRNAs used in this study did not match any *Nb* genomic sequences in public *Nb* genome data sources. Therefore, the genome-wide off-target effects of the SPIEM could not be addressed beyond the methylation at the sites adjacent to the target sequence.

### Different functions of NbAGO4A and NbAGO4B in spray-induced TGS

Despite over 85% sequence similarity between orthologs, only NbAGO4A was essential for silencing induction upon 24ntPro_U treatment. Functional divergence among orthologous AGO proteins in Solanaceae is not uncommon. For example, in tomato SlAGO2A but not SlAGO2B contributes to antiviral defense (Zhao et al., 2023), and NbAGO1A and NbAGO1B exhibit distinct developmental functions (Ludman et al., 2024). The critical function of NbAGO4 in RdDM has been demonstrated in the context of VIGS, but not in inverted repeat (IR)-induced RdDM. Overall, previous studies concluded that NbAGO4 shares similarities with AtAGO4 but is not functionally identical to it (Jones et al., 2006; Wang et al., 2019; Wang et al., 2020). Indeed, the protein sequence alignments show that NbAGO4A (formerly NbAGO4-2), NbAGO4B (formerly NbAGO4-1) (Wang et al., 2020), are the closest homologs of *AtAgo4* (**Figure 4A**) (Wang and Lozano-Duran, 2022). NbAGO4A and NbAGO4B proteins are structurally very similar and contain all major functional AGO domains, including PIWI, PAZ, and MID domains. According to AlphaFold models, the most striking difference between NbAGO4A and NbAGO4B structures lies in the disordered structure in the ArgoN domain (aa 140-170), despite high sequence similarity (**Supplementary Figure 6**). The ArgoN domain (spanning 90-180aa) has previously been linked to the miRNA-binding capacity of AGO1 (Xu et al., 2023). We therefore hypothesize that the structural differences in the ArgoN domain of NbAGO4B are critical for the HPST-siRNA-mediated TGS. In addition to structural features, *NbAgo4a* was shown to be expressed at approximately two-fold higher levels than *NbAgo4b* in the young leaves, the tissue which was sprayed (**Supplementary Table 2**) (Kurotani et al., 2023; Ranawaka et al., 2023). Considering the complete loss of TGS in *NbAgo4a* mutant, it is unlikely that the functional difference between the two orthologs is related to their expression levels.

### Implications of the 24nt siRNA spraying on epigenetic plant breeding

siRNAs are considered a key for epigenetic plant breeding (Chen, 2025). Our results show that it is possible to change the DNA methylation and histone methylation landscape by applying 24nt siRNAs exogenously. However, we also showed that the 24nt siRNA-induced silencing is temporary, which is an inhibitory aspect for employing this method in epigenetic plant breeding. Moreover, the promoter used in this study is CaMV-35S promoter, which may have a distinct regulation from endogenous promoters. Therefore, follow-up studies are required to show that promoter-targeting long dsRNAs spraying or 24nt siRNA spraying is sufficient for reducing endogene expression robustly. For that, an in-depth characterization of endogenous promoters will be required for *Nb*. It also remains unknown whether SPIEM applies to plants other than *Nb* for widespread agricultural use.

**Figure 5:**
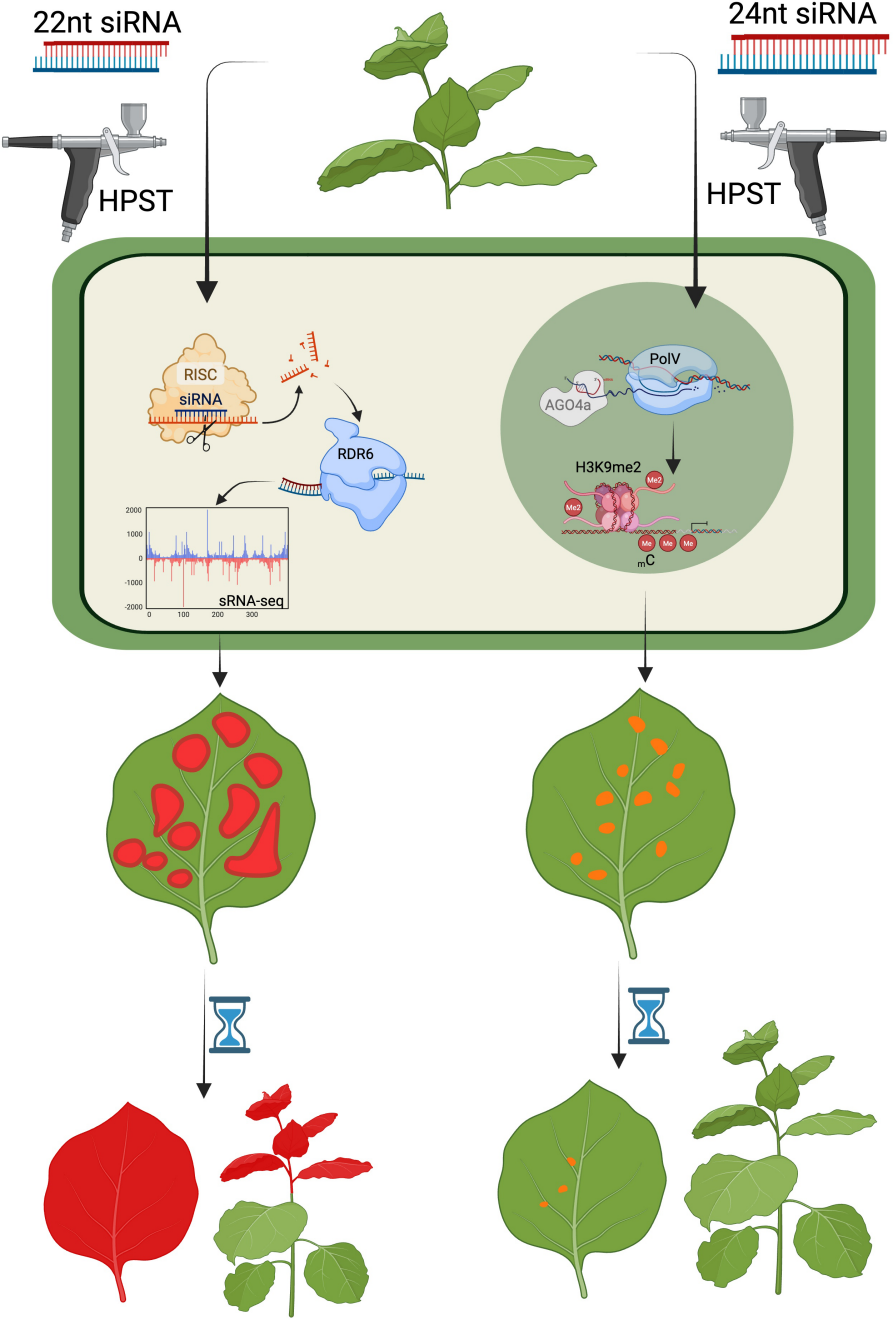
Graphical Summary: A comparison of exoRNA applications via 22nt siRNAs and 24nt siRNAs. While the HPST-mediated delivery of 22nt siRNAs targeting the gene body leads to Post Transcriptional Gene Silencing (PTGS), 24nt siRNAs targeting the promoter sequence by HPST induce transcriptional gene silencing (TGS). RISC and RDR6 play a critical role in PTGS. On the other hand, AGO4A (but not AGO4B) is the critical component controlling TGS in *Nb*. 22nt siRNA mediated silencing triggers a positive feedback loop via RDR6 and leads to systemic silencing in 16c plants, but 24nt siRNA mediated TGS dissolves over time due to leaf growth and lack of transitive RNA production. The figure is made by BioRender (2025).

In summary, we demonstrated for the first time that high-pressure spray delivery of a single 24nt siRNA is sufficient to induce targeted RdDM, H3K9me2 deposition, and TGS in an NbAGO4A-dependent manner (**Figure 5**). We also propose the demonstrated technique as a potential method for both studying the initiation of *de novo* RdDM and siRNA functional characterization.

Moreover, the ability to trigger stable epigenetic modifications via exogenous small RNAs highlights its potential for testing the effect of epitranscriptomic marks on the 24nt siRNAs, such as 2’-O-methylation, 5’-phosphorylation on TGS initiation.

## Data Availability

sRNA-seq libraries are available as NCBI GEO accessions, previously published libraries under GSE189777, and the 24ntProU datasets along with count-matrices for both datasets under GSE330610.

## Supplementary material

Supplementary Figure 1 24ntPro HPST induced 16c GFP silencing decays within weeks.

Supplementary Figure 2 16c GFP silencing upon application of siRNA-Carbon Dot complexes.

Supplementary Figure 3 Subdivided target gene regions highlight TGS vs. PTGs in sRNA-seq.

Supplementary Figure 4 sRNA sequencing of water-sprayed 16c samples.

Supplementary Figure 5 CaMV35S promoter Methylation patterns obtained from individual Sanger–seq reads.

Supplementary Figure 6 Comparison of NbAGO4A and NbAGO4b based on AlphaFold predictions.

Supplementary Table 1 siRNA sequences (dsRNAs displayed as individual strands).

Supplementary Table 2 GFP-Epi Allele distribution after selfing of 16c (het), 16cTGS (het) plants.

Supplementary Table 3 *NbAgo4a* and *NbAgo4b* expression level comparison in *Nb* LAB strain.
