# Supplementary material for: Exogenous 24nt siRNAs induce AGO4A-dependent silencing via promoter DNA methylation and H3K9me2 deposition
Source: Front Plant Sci. 2026 May 26;17:1826532. doi: 10.3389/fpls.2026.1826532 (PMC13246646; doi:10.3389/fpls.2026.1826532)
Supplement: Supplementary Figure 1 — 24ntPro HPST induced 16c GFP silencing decays within weeks. [file DataSheet1.docx]

**Supplementary Material**


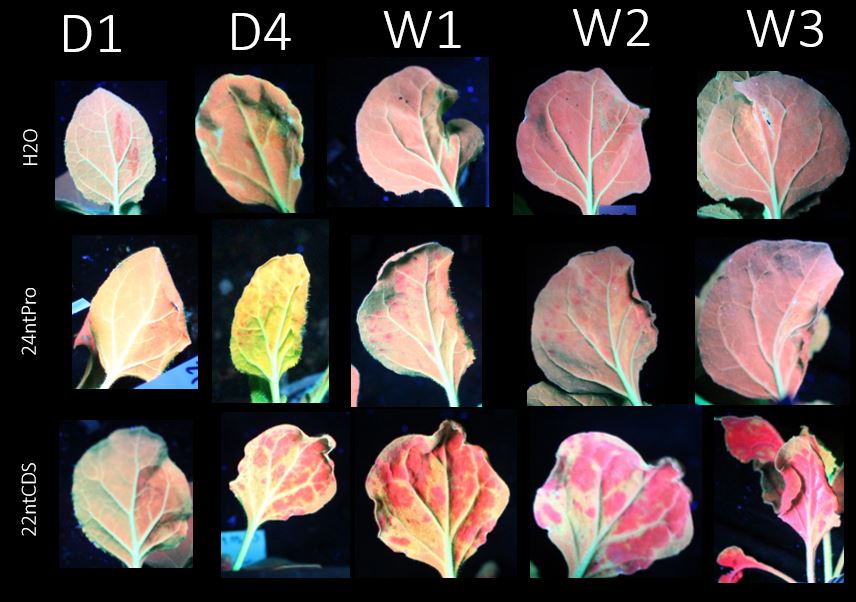


**Supplementary Figure 1: 24ntPro HPST induced 16c GFP silencing decays within weeks:**

Representative time series of leaves X days (D) or weeks (W) after HPST application. While 22ntCDS induced PTGS spreads over time as reported before, visible 24ntPro HPST induced GFP silencing peaks 7-10 days after application, thereafter becoming increasingly fainter. Note the temporary red hue of leaf sections damaged by the HPST shortly after HPST even in H2O controls, which quickly recovers.


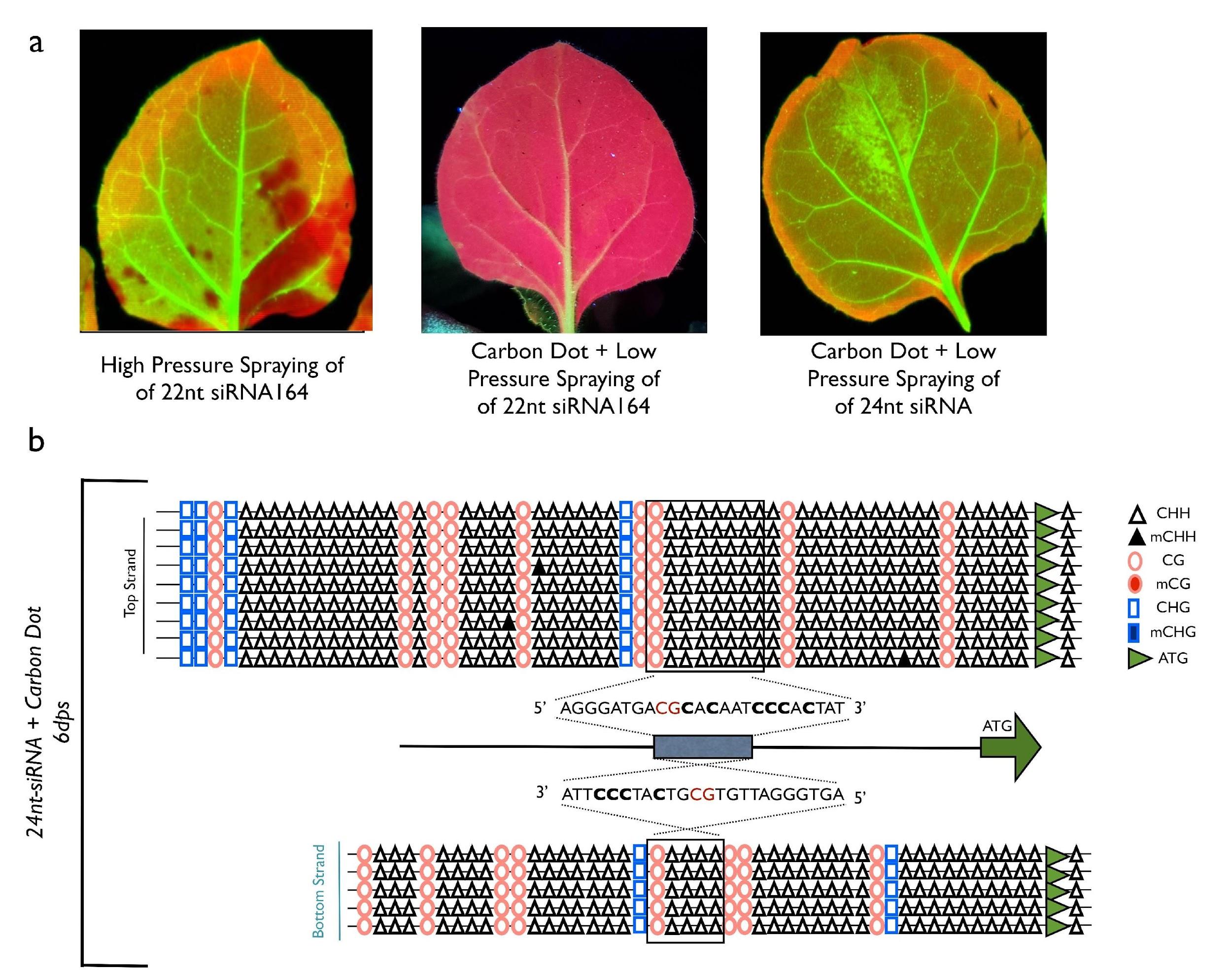


**Supplementary figure 2:** **16c GFP silencing upon application of siRNA-Carbon Dot complexes.**  The silencing phenotypes of 16C plants are shown. 22nt-siRNA164 by HPST (Uslu VV et al 2021) leads to consistent local silencing covering approximately 20% to 80% of the leaf surface. 22nt-siRNA164 by Carbon Dot and Low-Pressure Spraying (CD-LP) can lead to local silencing of the whole leaf area. However, no silencing upon 24nt-siRNA by CD-LP is observed in three independent experiments.


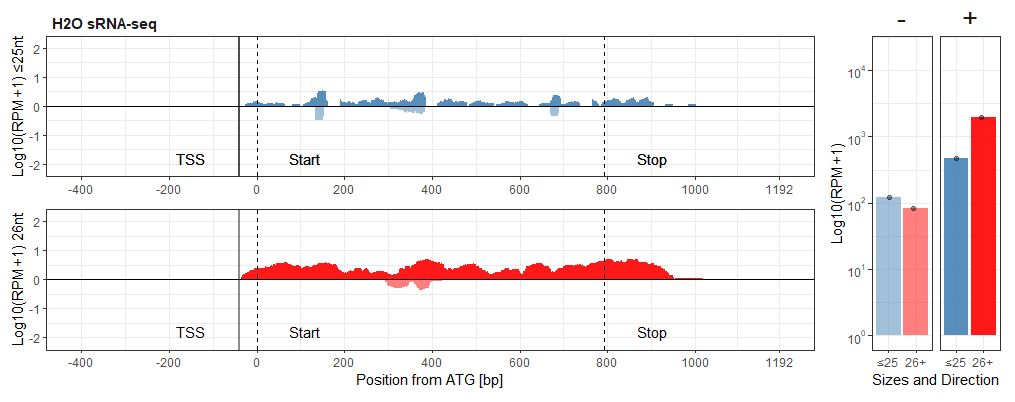


**Supplementary Figure 3:** **sRNA sequencing of water-sprayed 16c samples.** Reads per million (RPM) mapping to the *Nb* 16c GFP target transgene 460bp upstream and 1040bp downstream of start codon in sense (+) and antisense (-) direction, 6 days after water spraying HPST. CDS Start & Stop and transcription start site (TSS) indicated. The strand and size specific quantification of the reads are shown on the rightmost panel. in the rightmost panel. In each panel, red and blue signals above the X-axis indicate sense strand reads, whereas lighter-colored reads below the X-axis indicate anti-sense reads.


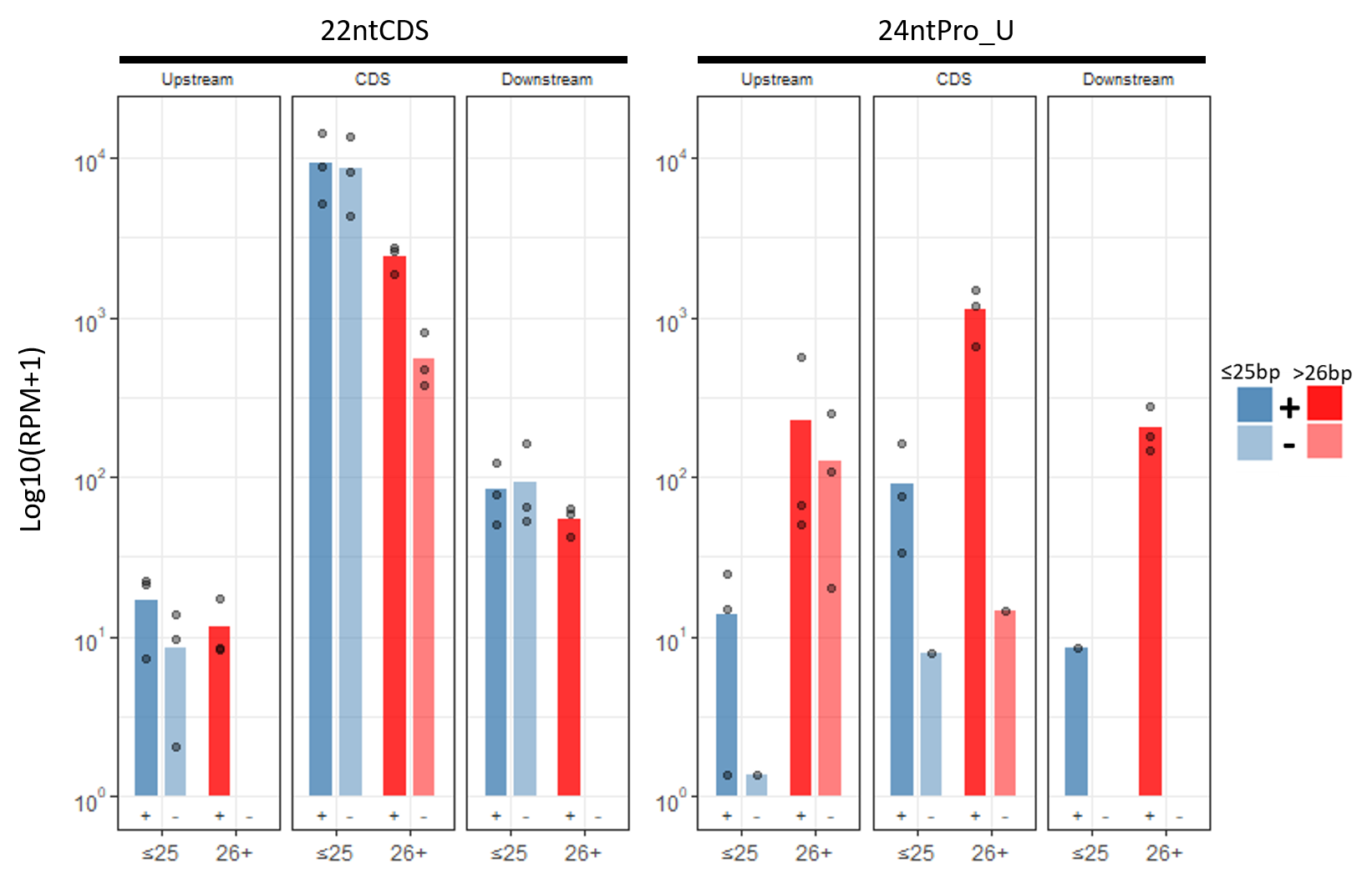


**Supplementary Figure 4: Subdivided target gene regions highlight TGS vs. PTGS in sRNA-seq.** sRNA seq Reads per million reads (RPM) mapping to different regions of 16c GFP target transgene 6 days after high-pressure spraying application of 22ntCDS data (Uslu et al., 2021) and 24ntPro_U. In the 22ntCDS treated libraries, abundant reads mapping mainly to the coding sequence (CDS) emerge in both sense and antisense direction, typical of PTGS- and RDR-dependent secondary RNA production as described in the previously published manuscript. 24ntPro_U treated samples, however contain only minimal antisense reads mapping to the CDS, but a strong emergence of reads longer than 25nt


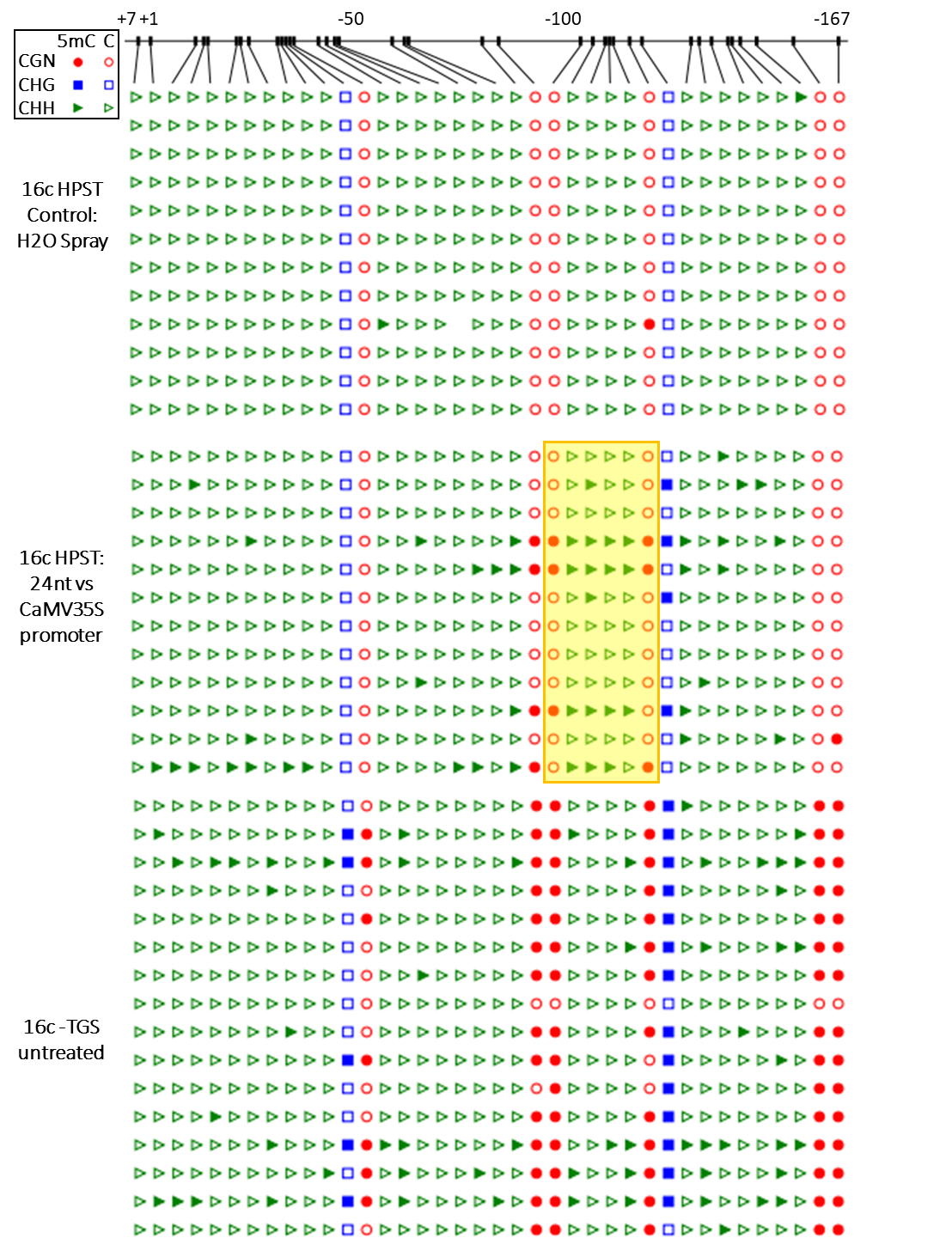


**Supplementary Figure 5: CaMV35S promoter Methylation patterns obtained from individual Sanger–seq reads**

Individual reads of the promoter target region, indicated as bases from ATG, amplified from bisulfite treated total DNA, then subcloned before Sanger-seq. Analysis and visualization using CyMATE (www.cymate.org). 16c mock spray and 16c -TGS mostly show low / high methylation throughout individual reads respectively, samples from 24nt siRNA treated 16c mostly contain sequences which are either barely, or highly methylated around the target sequence, not consistently an intermediate state as a mean value for methylation could imply.


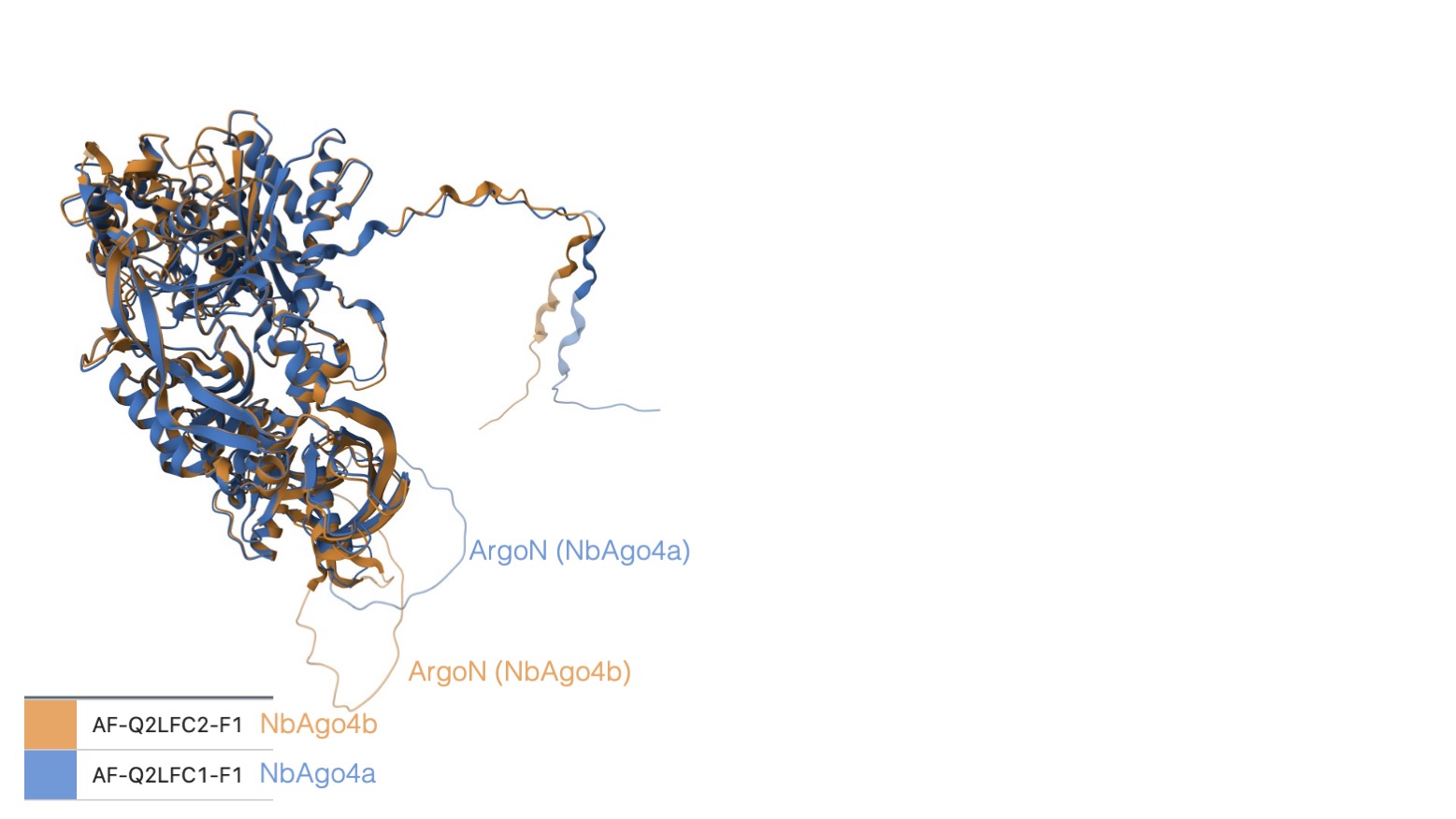


**Supplementary Figure 6: Comparison of NbAGO4A and NbAGO4b based on AlphaFold predictions.** Apart from minor structural changes in the critical domains, PAZ, Piwi, MID domains of NbAGO4A (shown in blue) and NbAgo4b (shown in orange) are almost fully overlapping. The most striking difference is in the disordered ArgoN domain of the AGO4 orthologs.
