# Supplementary material for: Exogenous 24nt siRNAs induce AGO4A-dependent silencing via promoter DNA methylation and H3K9me2 deposition
Source: Front Plant Sci. 2026 May 26;17:1826532. doi: 10.3389/fpls.2026.1826532 (PMC13246646; doi:10.3389/fpls.2026.1826532)
Supplement: Supplementary Table 1 — siRNA sequences (dsRNAs displayed as individual strands). [file Table1.docx]

Supplementary Tables

Supplementary Table 1: siRNA sequences (dsRNAs displayed as individual strands)

| Nucleotide name | Sequence 5’-3’ |
| --- | --- |
| 24ntPro_A | agggaugacgcacaaucccacuau |
| 24ntPro_A | agugggauugugcgucaucccuua |
| 24ntPro_U | uaagggaugacgcacaaucccacu |
| 24ntPro_U | ugggauugugcgucaucccuuacg |
| 22ntCDS | uuuccguauguugcaucaccuu |
| 22ntCDS | ggugaugcaacauacggaaaac |
| 22ntPro | augacgcacaaucccacuaucc |
| 22ntPro | auagugggauugugcgucaucc |
| 24ntCDS_138 | caaauuuucugucaguggagaggg |
| 24ntCDS_138 | cucuccacugacagaaaauuugua |

Supplementary Table 2: GFP-Epi Allele distribution after selfing of 16c (het) , 16cTGS (het) plants.

| 16c het x 16cTGS het | **Fully Silent plants (16cTGS hom)** | **GFP expressing plants (16c het x 16cTGS het or 16c hom)** |
| --- | --- | --- |
| Plant 1 | 34 | 74 |
| Plant 2 | 14 | 37 |
| Plant 3 | 25 | 80 |
| Plant 4 | 35 | 99 |
| Expected ratio | (1x) | (3x) |

Supplementary Table 3: NbAgo4a and NbAgo4b expression level comparison in Nb LAB strain (Kurotani et al., 2023).

| unit: TPM | **LAB_seed** | **LAB_root** | **LAB_leaf** | **LAB_stem** | **LAB_flower** |
| --- | --- | --- | --- | --- | --- |
| NbAgo4a | 22.6 | 160.16 | 33.82 | 79.99 | 53.62 |
| NbAgo4b | 13.88 | 42.22 | 18.57 | 26.91 | 24.86 |
